# The Caucasian Clover Gene *TaMYC2* Responds to Abiotic Stress and Improves Tolerance by Increasing the Activity of Antioxidant Enzymes

**DOI:** 10.3390/genes13020329

**Published:** 2022-02-10

**Authors:** Yihang Zhao, Yupeng Yang, Jingwen Jiang, Xiaomeng Zhang, Zewang Ma, Lingdong Meng, Guowen Cui, Xiujie Yin

**Affiliations:** College of Animal Science and Technology, Northeast Agricultural University, Harbin 150030, China; zhaoyh9712@163.com (Y.Z.); S210501072@neau.edu.cn (Y.Y.); jjw990824@163.com (J.J.); zhangxiaomeng20190@163.com (X.Z.); S210502008@neau.edu.cn (Z.M.); menglingdong0510@163.com (L.M.); cuigw603@126.com (G.C.)

**Keywords:** Caucasian clover, *TaMYC2*, abiotic stress, clone, antioxidant enzyme

## Abstract

Abiotic stress affects metabolic processes in plants and restricts plant growth and development. In this experiment, Caucasian clover (*Trifolium ambiguum* M. Bieb.) was used as a material, and the CDS of *TaMYC2*, which is involved in regulating the response to abiotic stress, was cloned. The CDS of *TaMYC2* was 726 bp in length and encoded 241 amino acids. The protein encoded by *TaMYC2* was determined to be unstable, be highly hydrophilic, and contain 23 phosphorylation sites. Subcellular localization results showed that *TaMYC2* was localized in the nucleus. *TaMYC2* responded to salt, alkali, cold, and drought stress and could be induced by IAA, GA_3_, and MeJA. By analyzing the gene expression and antioxidant enzyme activity in plants before and after stress, we found that drought and cold stress could induce the expression of *TaMYC2* and increase the antioxidant enzyme activity. *TaMYC2* could also induce the expression of ROS scavenging-related and stress-responsive genes and increase the activity of antioxidant enzymes, thus improving the ability of plants to resist stress. The results of this experiment provide references for subsequent in-depth exploration of both the function of *TaMYC2* in and the molecular mechanism underlying the resistance of Caucasian clover.

## 1. Introduction

Caucasian clover (*T. ambiguum* B.) is a perennial legume with a long crown [1,2]. This species is native to the cold Russian Caucasus, eastern Turkey, and northern Iran [3]. Caucasian clover can be cloned and propagated via rhizomes [4,5]. This species has strong adaptability and good resistance to drought, cold, and grazing [6]. Because of the harsh natural environment in which this species originated, the cold resistance of Caucasian clover is relatively strong [7]. Previously, our research group jointly used RNA-Seq and PacBio high-throughput sequencing technology to sequence the transcriptome of Caucasian clover and identified the genes related to the rhizome development [8]; this information in turn provided a rich genetic resource for the study of rhizome characteristics and the regulatory mechanism of stress resistance in Caucasian clover.

Abiotic stress such as drought, cold, and salt stress limits the growth and development, geographic distribution, yield, and quality of plants [9]. Abiotic stress affects the metabolic process of plants and limits the growth and development of plants at various stages [10,11,12]. Cold and drought are two common and critical abiotic stresses that can cause membrane damage, peroxidation, and accelerated senescence [13]. Plants employ complex physiological mechanisms for adapting and resisting abiotic stress [14]. Transcription factor (TF) families such as CBF [15], AP2, DREB [16], bHLH [17], and NAC [18] families, involved in this response, have also been gradually revealed. The expression of the genes encoding these TFs is often induced by abiotic stress, and these TFs also play an important regulatory role in plants.

bHLH TFs constitute the second major type of regulatory protein in plants [19]. These TFs have a specific bHLH domain, and they are involved in plant growth and development of plants and the response to abiotic stress [19]. MYC2 TFs are types of bHLH TFs and are important regulators of the jasmonic acid (JA) signaling pathway [20]. MYC2 TFs act as regulatory centers through the integration of multiple signaling pathways, which in turn affects various endogenous and exogenous signals that ultimately affect plant growth and development [21]. At the same time, these TFs participate in regulating the response of plants to abiotic stress [22]. Srivastava et al. found that *MYC2*s, which are component of the cytokinin signaling pathway, play an important role in the development of *Arabidopsis* seedlings [23]. Moreover, the MKK3–MPK6–MYC2 module positively regulates the biosynthesis and signal transduction of abscisic acid (ABA) in *Arabidopsis* [24], and *MYC2*s in *Brassica napus* regulate water loss and drought resistance by regulating stomatal opening and closing [25].

The use of plants with strong stress resistance (such as Caucasian clover) as research objects is effective for elucidating the comprehensive stress resistance mechanism in-depth to effectively improve and enhance plant stress resistance. However, research on the molecular mechanism underlying the stress resistance mechanism in Caucasian clover is lacking. Studies on *MYC2*s have mostly focused on JA signal transduction, but there are few studies related to abiotic stress [26]. In the present experiment, Caucasian clover was used as a material, and its *TaMYC2* gene was cloned. Bioinformatic analysis and expression pattern analysis data of *TaMYC2* in response to abiotic stress provide references for mining the function of *TaMYC2* and for studying the molecular mechanisms of plant stress resistance. *TaMYC2* responded to cold, drought, alkali, and salt stresses. *TaMYC2* expression was also induced by indoleacetic acid (IAA), gibberellin (GA_3_), and methyl jasmonate (MeJA). Lastly, *TaMYC2* overexpression in tobacco significantly improved tolerance to drought and cold.

## 2. Materials and Methods

### 2.1. Plant Materials, Growth Conditions, and Stress Treatments

Full, uniform seeds of Caucasian clover were collected and sown in vermiculite after the seed coats were polished with sandpaper. The seeds were cultivated at 25 °C under a 16 h photoperiod for 5 weeks, and Hoagland nutrient solution was applied every other day. Four Caucasian clover seedlings cultivated for 40 days were subjected to 4 °C, 150 mmol/L NaCl, 150 mmol/L NaHCO_3_, or 15% PEG-6000, and sampling was performed at 3 h, 6 h, 12 h, 24 h, and 48 h in each treatment. Other Caucasian clover seedlings were sprayed with 100 µmol/L IAA, GA_3_, and MeJA, and samples were taken at 3 h, 6 h, 12 h, and 24 h in each treatment. Untreated plant materials of the same growth stage were used as controls (CKs). Each treatment involved three replications. Samples were taken from the roots, stems, and leaves of plants in each treatment, wrapped in aluminum foil, flash-frozen in liquid nitrogen, and then stored at −80 °C.

### 2.2. Cloning of the TaMYC2 Gene and Quantitative Real-Time PCR (qRT–PCR) Analysis of Gene Expression

Total RNA was extracted using an Ultrapure RNA Kit (CWBIO, Taizhou, China). The cDNA template for reverse transcription PCR was synthesized using HiScript II Reverse Transcriptase (Vazyme, Nanjing, China). The coding DNA sequence (CDS) of *TaMYC2* was obtained according to the results of transcriptome sequencing, and the primers used for cloning were designed using Primer 5 software (Table S1). Based on the cDNA template, 2× Taq Master Mix (Vazyme, Nanjing, China) was used for PCR amplification. The PCR product was electrophoresed on a 1% agarose gel, and the target fragment was recovered with a FastPure Gel DNA Extraction Mini Kit (Vazyme, Nanjing, China). The recycled product was cloned into a pCE2 TA/Blunt-Zero vector (Vazyme, Nanjing, China) and sequenced for confirmation.

With cDNA used as a template, real-time fluorescence quantification was performed using internal reference and fluorescence quantification primers (Table S1). All the qRT–PCR analyses were performed using a ChanQ Universal SYBR qPCR Master Mix Kit according to the manufacturer’s instructions, and the relative gene expression was calculated using the 2^−ΔΔCt^ method.

### 2.3. Bioinformatic Analysis

The NCBI BLASTP program was used to search for homologous sequences of *TaMYC2*. DNAMAN was then used to compare the amino-acid sequence of *TaMYC2* with those of other species. A phylogenetic tree was constructed using ClustalX 2.1 and MEGA 5. Domain prediction of *TaMYC2* was carried out by SMART. The physicochemical properties, hydrophilicity, and phosphorylation sites of the amino acids were analyzed by ProtParam, ProtScale, and NetPhos 3.1.

### 2.4. Construction of TaMYC2 Overexpression and Transient Expression Vectors in Tobacco

Overexpression and instantaneous-expression primers (Table S1) were used for PCR and gel recovery to obtain the inserted fragments. pCAMBIA1300-35S-sGFP plasmids were linearized by double digestion with *Bam*HI and *Sac*I (Figure S1). Overexpression and instantaneous-expression vectors were obtained by recombination of the inserted fragments and vectors, respectively. The vectors were transferred into *Escherichia coli* DH5α and cultured for 12 h. Several individual clones were selected for use in PCR detection and sequence comparison. The plasmid extracted from the bacterial solution and with the correct sequencing result was transformed into Agrobacterium EHA105 by the freeze–thaw method, after which the bacteria were spread onto double-resistant YEB media that included kanamycin and rifampin for selection. After 60 h of culture, a few individual colonies were selected for PCR verification.

### 2.5. Subcellular Localization

Seeds of *Nicotiana benthamiana* were sown in a mixture of vermiculite and potting soil (1:3). Each tobacco plant was then transplanted into a separate pot after the seeds germinated and was watered every day. A solution of transformed bacteria (after being cultured for 1 month) was diluted with buffer solution and then injected into the abaxial side of tobacco leaves with a needle. The infected leaf tissue was obtained after the plants were placed in the dark for 2 days. The leaf sections were observed with a confocal laser microscope.

### 2.6. TaMYC2-Overexpressing Tobacco

Sterilized *Nicotiana tabacum* L. ‘K326′ seeds were transferred to culture media. After the seeds had germinated and grown for 28 days, their leaves were taken as explants for preculture. Agrobacterium cells containing the plasmid with the target gene were cultured to an appropriate concentration and then used for infection and cocultivation of explants. The cocultured tobacco leaves were induced to form calli, and rooting was induced after two hygromycin-based selections. Then, the obtained plants were subjected to PCR to detect and obtain T_0_ tobacco plants. Eight transgenic lines were successfully identified, and three of them were selected for subsequent experiments (Figure S2). These three transgenic tobacco plants were transplanted into soil and cultivated until they produced seeds, which were harvested. The harvested seeds were placed in media that included hygromycin to germinate for selection, and the germinated seedlings were subjected to PCR detection to obtain T_1_-transformed *TaMYC2-*overexpressing tobacco plants, which were transplanted into soil for cultivation.

### 2.7. Physiological Measurements

*TaMYC2-*overexpressing tobacco and wild-type (WT) tobacco cultivated for 28 days were cultivated at 4 °C for 7 days to simulate cold stress and continuously irrigated with 100 mL of 15% PEG-6000 to simulate drought stress for 7 days. The leaves of plants exposed to CK, cold, and drought-stress conditions were sampled from the WT and three transgenic lines (L1, L2, and L3). There were three biological replicates of each WT, L1, L2, and L3 plant. Malondialdehyde (MDA) accumulation was measured using the thiobarbituric acid-based method [27], and the proline (Pro) content was determined by the acid ninhydrin method [27]. The chlorophyll content was measured as described by Feng et al. [28]. For the enzyme activity assays, the superoxide dismutase (SOD) activities were determined by measuring the inhibition rate of the enzyme to O_2_^−^ produced by xanthine morpholine via xanthine oxidase; an SOD kit (Keming, Suzhou, China) was used for this. The catalase (CAT) activity was measured on the basis of the degradation of H_2_O_2_ at 405 nm via a CAT kit (Keming, Suzhou, China). Similarly, the peroxidase (POD) activity was measured on the basis of the change in absorbance at 420 nm caused by catalysis of H_2_O_2_; a POD kit (Keming, Suzhou, China) was used for this. Three technical replications were included for all index determinations.

### 2.8. Statistical Analysis

Excel 2019 was used to organize the data and create column charts. One-way analysis of variance was performed via SPSS 22.0, and Duncan’s method was used for multiple comparisons.

## 3. Results

### 3.1. Cloning of the TaMYC2 Gene from Caucasian Clover

Total RNA of Caucasian clover was extracted (Figure S3a) and reverse-transcribed into cDNA. The quality of cDNA was verified using internal reference gene primers (Table S1, Figure S3b). Forward and reverse primers for cloning the CDS of *TaMYC2* were designed according to the transcriptomic data, (Table S1). RT-PCR technology was used to amplify the CDS of *TaMYC2* (Figure S3c), and the amplified products were cloned into a T vector following gel recovery. Sequencing revealed that the CDS of *TaMYC2* was 726 bp in length and encoded 241 amino acids (Table S2).

### 3.2. Bioinformatic Analysis of TaMYC2

**Figure 1:**
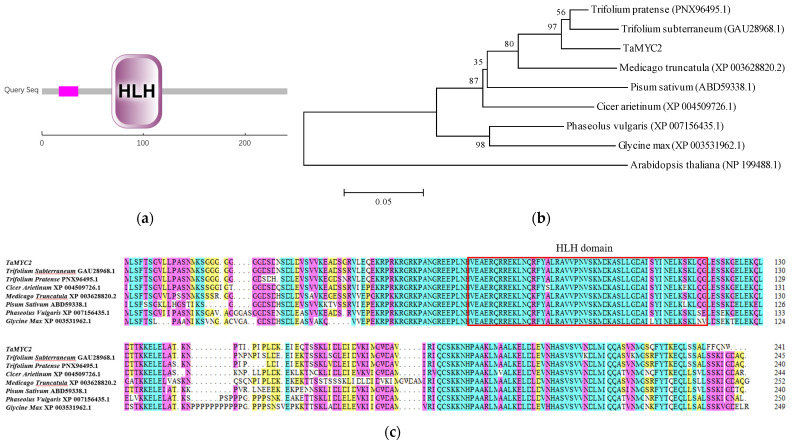
Bioinformatic analysis of *TaMYC2* in Caucasian clover. (**a**) SMART domain analysis. (**b**) Phylogenetic analysis of *TaMYC2* in Caucasian clover. (**c**) Multispecies homologous amino-acid sequence alignment.

The amino-acid sequence of *TaMYC2* was uploaded to the SMART website for domain prediction, and the results showed that *TaMYC2* contains an HLH domain located between residues 69 and 118 (Figure 1a). ProtParam was used to analyze the physicochemical properties of the *TaMYC2* peptide: The molecular weight of C_1160_H_1907_N_333_O_368_S_8_ was 26663.39 KD, and the isoelectric point was 7.77. The most abundant amino acid was leucine, which reached 11.2%, and the least abundant was cysteine at only 0.4%. The amino-acid sequence of *TaMYC2* comprises 34 negatively charged aspartic acid and glutamic acid residues and 35 positively charged arginine and lysine residues. The protein was considered unstable (instability index 38.42), and the lipid index was 87.80. The amino-acid sequence of *TaMYC2* was analyzed by NCBI Protein BLAST and compared with those of *Trifolium pratense*, *Medicago truncatula*, *Arabidopsis thaliana*, and *Cicer arietinum* by DNAMAN (Figure 1b). A phylogenetic tree was constructed by MEGA 5, which revealed that TaMYC2 was most closely genetically related to the MYC2 protein from red clover (Figure 1c).

The phosphorylation sites of *TaMYC2* were predicted using the NetPhos 3.1 website (Figure S4a). There were a total of 23 phosphorylation sites, including 19 phosphorylation sites involving serine, three phosphorylation sites involving threonine, and one phosphorylation site involving tyrosine. Analyses of the hydrophilicity and hydrophobicity of the *TaMYC2* protein were performed via the ProtScale website. It can be seen from the figure that most amino acids have negative values, and many negative amino acids have strong hydrophilic properties. Therefore, it can be inferred that *TaMYC2* is a hydrophilic protein (Figure S4b). Moreover, transmembrane domain analysis showed that the *TaMYC2* protein does not contain any transmembrane domains (Figure S4c). Additionally, *TaMYC2* is not a secreted protein and contains no signal peptide (Figure S4d).

### 3.3. Expression Analysis of TaMYC2 in Response to Abiotic Stress

**Figure 2:**
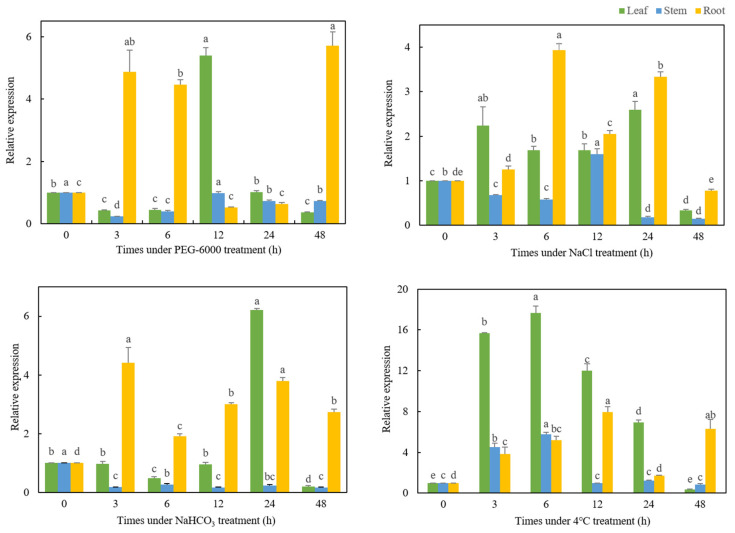
Expression analysis of *TaMYC2* in Caucasian clover under abiotic stress. The different lowercase letters indicate significant differences under different treatment times for the same tissue (*p* < 0.05). The same scheme applies to the figures shown below.

The expression of *TaMYC2* changed in response to drought, salt, alkali, and cold stress (Figure 2). Under 15% PEG-6000-simulated drought stress, the variation of *TaMYC2* expression in different plant tissues tended to be different. The expression level of *TaMYC2* in the roots of PEG-6000-treated plants was significantly higher than that of the CK plants (*p* < 0.05), but the expression level of *TaMYC2* in the stems and leaves significantly decreased (*p* < 0.05) after PEG treatment for 3 h and 6 h. After PEG treatment for 12 h, the gene expression in the leaves reached the highest level, and the gene expression in the stems increased to the same level as that in the CK, but the gene expression in the roots significantly decreased compared with that at 3 h and 6 h (*p* < 0.05). After PEG treatment for 48 h, the expression of *TaMYC2* in the leaves was the lowest, and the expression of *TaMYC2* in the roots was the highest.

Under 150 mmol/L NaCl stress, the expression of *TaMYC2* in the leaves and roots of the treated plants was significantly higher than that in the CK (*p* < 0.05), and the expression of *TaMYC2* in the stems was significantly lower than that in the CK after 3 h (*p* < 0.05). The expression level of *TaMYC2* in the stems further decreased after 6 h of NaCl treatment, while the expression level of *TaMYC2* in the roots further increased to a level that was significantly more than that in the other treatments (*p* < 0.05). After 12 h of NaCl treatment, the gene expression levels in all the evaluated tissues significantly increased compared with the levels of the CK (*p* < 0.05), and the expression level in the stems was significantly higher than that in the other treatments (*p* < 0.05). The expression of *TaMYC2* in the leaves was significantly higher after 24 h of NaCl treatment than after the other treatments (*p* < 0.05), and the expression of *TaMYC2* in the leaves of plants after 48 h of NaCl treatment was significantly lower than that of the CK plants (*p* < 0.05).

Under 150 mmol/L NaHCO_3_ alkali stress, the expression level of *TaMYC2* in the roots of the plants in the five treatment groups was significantly higher than that in the CK (*p* < 0.05), and the highest expression level was detected after 3 h of treatment. The expression level of *TaMYC2* in the stems of the treated plants was significantly lower than that of CK plants (*p* < 0.05). Among the plants in the five treatment groups, the expression level of *TaMYC2* in the leaves of the plants in the 24 h group was significantly higher than that in the CK group (*p* < 0.05).

Under cold stress, with increasing stress duration, the expression of *TaMYC2* in the three tissues first increased and then decreased. Under 4 °C for 3 h and 6 h, the expression of *TaMYC2* in the three tissues was significantly higher than that of the CK (*p* < 0.05), and the gene expression in the leaves and stems peaked when the plants were exposed 4 °C for 6 h. The expression of *TaMYC2* in the roots peaked when the plants were exposed to 4 °C for 12 h and was significantly higher than that in the other treatments (*p* < 0.05), while the expression of *TaMYC2* in the leaves and stems gradually decreased beginning at 12 h, reaching the lowest level at 48 h.

### 3.4. Analysis of TaMYC2 Expression Patterns in Response to Hormone Treatments

**Figure 3:**
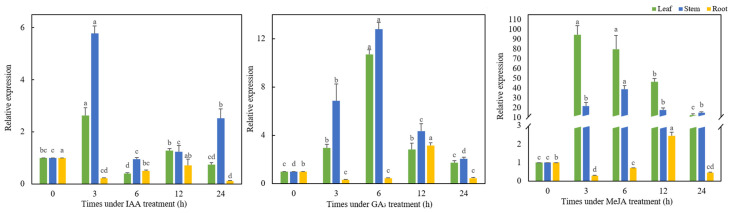
Analysis of *TaMYC2* expression patterns in response to various hormone treatments.

The expression level of *TaMYC2* in response to IAA, GA_3_, and MeJA treatments changed drastically, and the change trends under the three treatments were different (Figure 3). The expression of *TaMYC2* in the stems and leaves under the 3 h IAA treatment was significantly higher than that under the other treatments (*p* < 0.05), while the expression in the roots in the treated plants was significantly lower than that in the CK plants (*p* < 0.05). The expression of *TaMYC2* in the stems and leaves was the lowest when IAA treatment was applied for 6 h. The expression of *TaMYC2* in the roots decreased to the lowest value after IAA treatment for 24 h, which was significantly lower than that of the CK (*p* < 0.05). Under GA_3_ treatment, the expression of *TaMYC2* in the leaves and stems first increased and then decreased with increasing treatment duration. The expression levels of *TaMYC2* in the leaves and stems were the highest upon treatment for 6 h, the levels of which were approximately 10 and 12 times that of the CK, respectively. Except at 12 h, the expression of *TaMYC2* in the roots of the GA_3_-treated plants was significantly lower than that of the CK plants at each treatment time (*p* < 0.05). Under MeJA treatment, the expression of *TaMYC2* in the leaves and stems of the treated plants increased first and then decreased with increasing treatment time, and the expression of *TaMYC2* in the leaves and stems of plants at each treatment time was significantly higher than that of the CK plants (*p* < 0.05). The expression of *TaMYC2* in the leaves was the highest at 3 h, the level of which was approximately 90 times that of the CK, and the expression of *TaMYC2* in the stems was the highest at 6 h, the level of which was approximately 40 times that of the CK. The expression of *TaMYC2* in the roots was the lowest after 3 h of treatment with MeJA and was significantly lower than that of the CK plants (*p* < 0.05), but then *TaMYC2* expression increased to the highest value at 12 h treatment with MeJA, the level of which was significantly higher than that of the CK (*p* < 0.05).

### 3.5. The TaMYC2 Protein Localizes to the Nucleus

**Figure 4:**
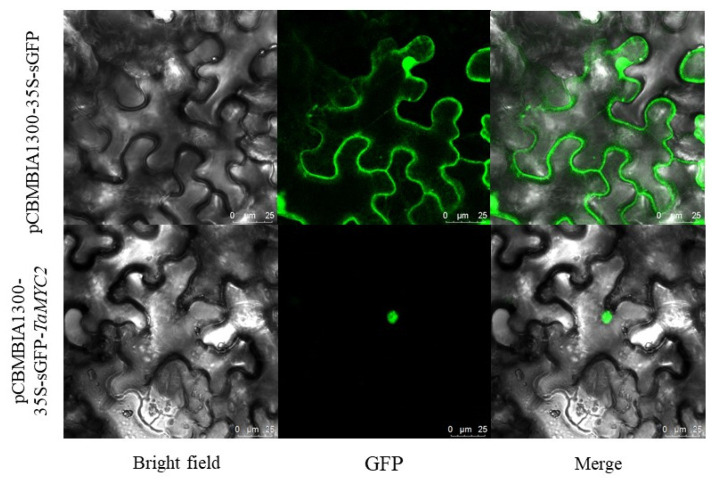
Subcellular localization of *TaMYC2* in tobacco.

pCBMBIA1300-35S-sGFP plasmids were digested with *Bam*HI and *Sac*I, and the vectors were linearized. The target fragment was amplified with transient-expression primers using the pCE2-*TaMYC2* plasmid as a template, and the tobacco transient expression vector p1300-sGFP-*TaMYC2* was obtained after recombination. The resulting tobacco transient expression vector construct was transformed into Agrobacterium EHA105, and the transformed cells were injected into tobacco. The expression position of *TaMYC2* was observed via confocal laser microscopy, and the results showed that *TaMYC2* was expressed in the nucleus (Figure 4).

### 3.6. Functional Verification of TaMYC2-Overexpressing Tobacco in Response to Abiotic Stress

**Figure 5:**
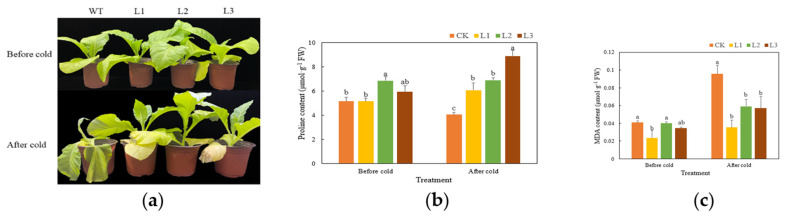

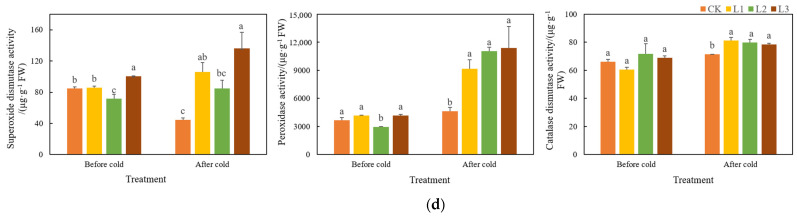
Functional verification of *TaMYC2-*overexpressing tobacco in response to cold stress. The different lowercase letters indicate significant differences under the different treatments for the same plant (*p* < 0.05); the same scheme applies to the figures below. Comparisons of phenotypes (**a**), MDA contents (**b**), Pro contents (**c**), and antioxidant enzyme activities (**d**) between WT and transgenic tobacco under cold stress.

After 7 days of being exposed to 4 °C, transgenic plants were growing well, and there was no obvious change in phenotype compared with that of the previous treatment. However, the WT plants showed obvious wilting (Figure 5a). Under the same cold stress, the MDA content of the WT plants increased significantly, although the increase in MDA content of the transgenic plants was lower. The MDA content of the transgenic plants was significantly lower than that of the WT plants (*p* < 0.05), and the MDA content of the WT plants was approximately twice that of the transgenic plants (Figure 5b). The Pro content of the WT plants decreased under cold stress, while the Pro content of the transgenic plants increased slightly. The Pro content of the transgenic plants was significantly higher than that of the WT plants (*p* < 0.05) (Figure 5c). Under cold stress, the SOD, CAT, and POD activities of the transgenic plants were significantly higher than those of the WT plants (*p* < 0.05) (Figure 5d). The SOD activity in the WT plants under cold stress decreased, while the SOD activity in the transgenic plants increased. Under cold stress, the POD activity in the WT plants increased slightly, while the POD activity in the transgenic plants increased significantly, reaching a level twice that of the WT plants. Lastly, the CAT activity in the WT plants and transgenic plants increased under cold stress, but the CAT activity in the transgenic plants increased to a greater degree.

**Figure 6:**
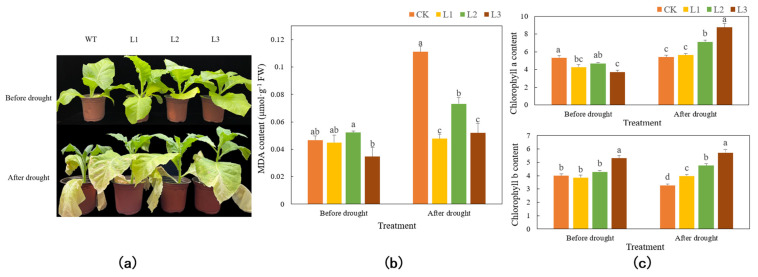

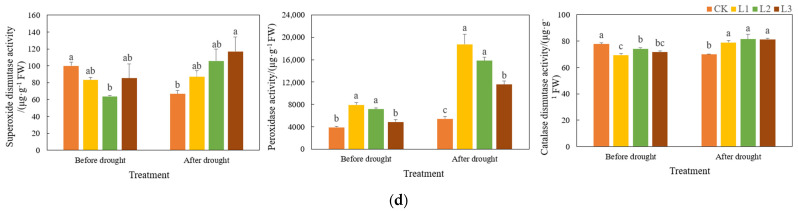
Functional verification of *TaMYC2-*overexpressing tobacco under drought stress. Comparisons of phenotypes (**a**), MDA contents (**b**), chlorophyll contents (**c**), and antioxidant enzyme activities (**d**) between WT and transgenic tobacco under drought stress.

After 15% PEG-6000 treatment for 7 days, the leaves of both the WT plants and the transgenic plants were withered and wilted, but the wilting of the WT plants was more severe than that of the transgenic plants. At the same time, the growth of the WT plants was also suppressed, and the plant height and leaf size were obviously lower than those of the transgenic plants (Figure 6a). After 7 days of drought treatment, the content of chlorophyll *a* in the WT plants was approximately the same, while the content of chlorophyll *b* decreased. However, the chlorophyll *a* content of the transgenic plants greatly increased. Under drought stress, the chlorophyll *a* and chlorophyll *b* contents of the transgenic plants were significantly higher than those of the WT plants (*p* < 0.05) (Figure 6b). In addition, under drought stress, the MDA content of the WT plants more than doubled, while the MDA content of the transgenic plants only slightly increased compared to the levels before the stress. The MDA content of the transgenic plants was significantly lower than that of the WT plants (*p* < 0.05) (Figure 6c). Moreover, under drought stress, the SOD, CAT, and POD activities of the transgenic plants were significantly higher than those of the WT plants (*p* < 0.05) (Figure 6d). However, after drought stress, the SOD activity in the WT plants decreased, while the SOD activity in the transgenic plants increased significantly. The POD activity in the WT plants increased, but the degree of increase in the transgenic plants was greater, approximately two to four times that of the WT plants. After drought stress, the CAT activity in the WT plants decreased, while the CAT activity in the transgenic plants increased, but the difference in activity between the two types was relatively small.

### 3.7. Expression Analysis of Reactive Oxygen Species (ROS) Scavenging-Related and Stress-Responsive Genes in Transgenic and WT Plants before and after Treatment

**Figure 7:**
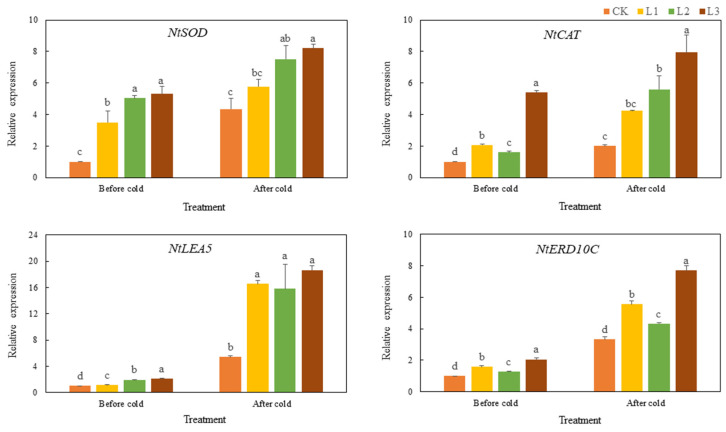
Analysis of the relative expression of ROS scavenging-related and stress-responsive genes under in response to stress.

**Figure 8:**
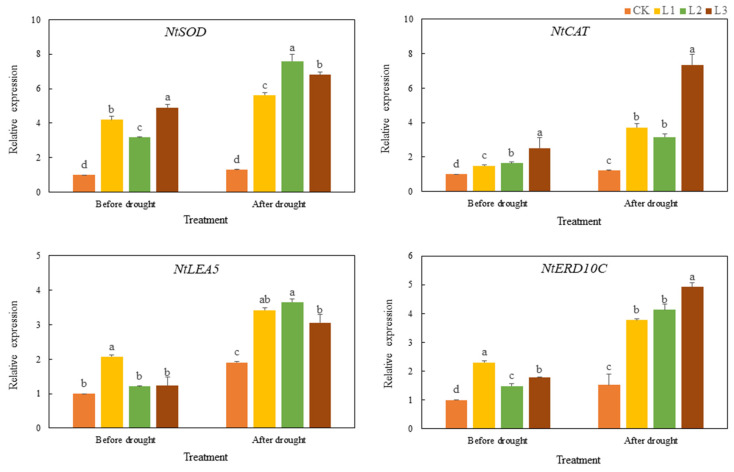
Analysis of the relative expression of ROS scavenging-related and stress-responsive genes in response to drought stress.

After cold stress, the expression levels of *NtSOD*, *NtCAT*, *NtLEA5*, and *NtERD10C* in the WT tobacco and transgenic tobacco increased, but the gene expression level in the transgenic tobacco increased to a greater degree (Figure 7). Before and after cold stress, the expression of ROS scavenging-related and stress-responsive genes in the transgenic plants was significantly higher than that in the CK plants (*p* < 0.05). This indicates that the overexpression of *TaMYC2* likely increases the induction of ROS scavenging-related and stress-responsive genes under cold stress. The expression changes of ROS scavenging-related and stress-responsive genes in WT tobacco and *TaMYC2-*overexpressing tobacco before and after drought stress were similar to those before and after cold stress, respectively (Figure 8). Overexpression of *TaMYC2* enhanced the induction of ROS scavenging-related and stress-responsive genes in response to drought stress. Drought stress also induced the expression of ROS scavenging-related and stress-responsive genes. However, the degree of induction of ROS scavenging-related and stress-responsive genes in the transgenic plants was higher than that in the WT plants.

## 4. Discussion

Bioinformatic technology can be used to analyze the sequence of genes, determine the functions of genes, and improve the efficiency of genetic research [29]. In this study, RT-PCR was used to successfully clone the CDS of the Caucasian clover *MYC2* gene according to transcriptomic data. The sequencing results after cloning were consistent with the predicted transcriptomic results, and the gene was named *TaMYC2*. The full-length CDS of *TaMYC2* was determined to be 726 bp, encoding 241 amino acids. The MYC2 TF is a bHLH-type TF containing a bHLH domain [30,31]. The conserved bHLH domain consists of an alkaline region and the HLH region; the HLH region is located at the C-terminus of the bHLH domain, while the alkaline region is located at the N-terminus of the bHLH domain [32,33]. SMART domain analysis showed that the *TaMYC2* gene has an HLH domain, which showed that the *TaMYC2* gene belongs to the MYC2 TF family. The bHLH domain is mainly composed of hydrophobic residues that form two amphiphilic α helices, which also confirms the supposition that *TaMYC2* is a hydrophobic protein according to the ProtScale website. MYC TFs are ubiquitous in plants. Our phylogenetic tree showed that *TaMYC2* is highly homologous to MYC2 in red clover (which is in the same genus) and *M. truncatula*, which is also in the legume family, but that *TaMYC2* has homology with MYC2 in *A. thaliana*.

Phytohormones are important regulators in plants and are highly important at all stages of plant growth and development [34]. MLC TFs are widespread in plants and animals and have a variety of regulatory functions, such as in JA signaling in plants [35]. By transforming *ZmMYC2* from maize into *A. thaliana*, Jing Yefu et al. found that *ZmMYC2* interacts with JA signal repressors, revealing that *ZmMYC2* plays various regulatory roles in JA and auxin signal transduction in maize [36]. Studies have shown that MYC2 TFs are involved in the regulation of signal transduction involving ABA, JA, and other hormones and play important roles in plant growth and development [37]. Moreover, studies have shown that JA and ABA can induce the expression of *MYC2*s [38]. In the present study, we found that *TaMYC2* expression in plant leaves and stems increased significantly in response to three exogenous hormones, while gene expression in the roots was induced to a lesser degree. This indicates that *TaMYC2* in Caucasian clover leaves and stems is relatively sensitive to exogenous application of these three hormones. The expression pattern of *TaMYC2* in the roots of Caucasian clover plants was quite different from the expression pattern in the leaves and stems. We speculate that this difference is due to differences in the regulatory mechanisms of plant hormones and to the low expression of the *TaMYC2* gene in the roots. When GA_3_ and MeJA were applied exogenously, the expression of *TaMYC2* in the Caucasian clover leaves and stems increased first and then decreased, which was consistent with the *MYC2* expression pattern reported by Wang et al. in rape in response to exogenous application of ABA and JA [38]. Moreover, GA_3_ and MeJA within a certain period after treatment could induce the expression of *TaMYC2* in Caucasian clover leaves and stems. Thus, *TaMYC2* is not only involved in hormone signal transduction but also regulated by exogenous hormones.

Salt–alkaline stress is an important and common abiotic stress that can cause water stress, ion toxicity, and even plant death [39,40]. Deepanjali Verma et al. found that salt stress can activate *MYC2*s through the mitogen activated protein kinase (MAPK) cascade pathway and that *MYC2*s regulate the synthesis of Pro by regulating *P5CS1* expression [41]. In the present study, salt stress induced the expression of *TaMYC2* in the leaves and roots but inhibited its expression in the stems. Similarly, alkaline stress induced the expression of *TaMYC2* in the roots but inhibited its expression in the stems. These results show that *TaMYC2* responds differently to salt stress and alkali stress, which is especially true in the tissues whose sensitivity to the various stresses differed.

Drought stress inhibits the expansion of leaves, causes stomata to close, and reduces the photosynthetic rate and enzyme activities of plants [42,43]. Cold stress can severely affect the normal growth and development of plants and cause disorders involving plant metabolism. Cold stress can also disrupt the water balance in plants and destroy plant membrane systems [44]. In the present study, analysis of gene expression patterns revealed that *TaMYC2* responds to 4 °C and polyethylene glycol (PEG) stress; thus, drought and cold stress can affect the expression of *TaMYC2*. Drought stress had a greater impact on the expression of *TaMYC2* in the plant roots, while the impact in the stems and leaves was relatively small. Under cold stress, the expression of *TaMYC2* in the three parts showed a trend of increasing first and then decreasing. This change trend was more consistent with the change trend of SOD and POD activity in plants under cold stress [45]. SOD, POD, and CAT are important antioxidant enzymes in plants that are involved in resistance to adversity and remove active oxygen [46,47]. Therefore, *TaMYC2* may participate in the regulation of plant protection-related mechanisms, promote the production of plant protection-related enzymes, and enhance the ability of plants to resist low temperature stress.

In addition to the three common antioxidant enzymes mentioned above, MDA, Pro, chlorophyll *a*, and chlorophyll *b* are important indicators to evaluate the tolerance of plants to cold and drought stress. Plant cell membranes can become disrupted by abiotic stress, and membrane permeability increases. The continuous accumulation of active oxygen eventually leads to the peroxidation of membrane lipids, resulting in the production of MDA [48,49]. Plants resist abiotic stress by increasing the content of osmotic adjustment substances such as Pro [50]. By measuring the physiological indicators of WT and transgenic tobacco under cold and drought stress, we found that the *TaMYC2*-overexpressing plants presented a lower degree of membrane lipid peroxidation and higher antioxidant enzyme activity than the WT plants did. Moreover, the transgenic plants had higher chlorophyll *a* and chlorophyll *b* contents under drought stress, as well as higher Pro contents under cold stress. These results indicate that *TaMYC2-*overexpressing plants have better resistance to abiotic stress than WT plants do. This also confirmed our previous hypothesis that *TaMYC2* enhances plant resistance to stress by promoting the production of plant protection enzymes. The overexpression of *TaMYC2* in response to abiotic stress could regulate antioxidant enzyme activity in plants and reduce the degree of peroxidation of plant membrane lipids.

Lastly, the expression of ROS scavenging-related and stress-responsive genes was measured in transgenic tobacco under drought and cold stress. Studies have shown that these genes or their homologous genes play roles in ROS scavenging- and stress-related responses [51,52,53,54]. Overexpression of *TaMYC2* could induce the expression of ROS scavenging-related and stress-responsive genes to a certain extent. At the same time, overexpression of *TaMYC2* could increase the expression of ROS scavenging-related and stress-responsive genes under cold and drought stress, thereby enhancing the ability of the transgenic plants to respond to stress. On the basis of the above conclusions, we speculated that *TaMYC2* was highly responsive to abiotic stresses such as cold and drought. Moreover, overexpression of *TaMYC2* could help regulate antioxidant enzyme activity in plants and reduce the degree of peroxidation of plant membrane lipids. At the same time, *TaMYC2* overexpression induced the expression of ROS scavenging-related and stress-responsive genes, enhanced the ability of plants to respond to stress, and increased antioxidant enzyme activities, thereby improving plant stress resistance.

## 5. Conclusions

In this experiment, the CDS of *TaMYC2* of Caucasian clover was successfully cloned. After it was sequenced, the CDS of the gene was determined to be 726 bp in length, encoding 241 amino acids. The protein encoded by *TaMYC2* contains an HLH domain and was most closely phylogenetically related to the MYC2 protein from red clover. The protein encoded by *TaMYC2* is unstable, highly hydrophilic, and contains 23 phosphorylation sites. Subcellular localization results showed that *TaMYC2* was localized in the nucleus. *TaMYC2* is expressed in response to salt, alkali, cold, and drought stress and is induced by three hormones, i.e., IAA, GA_3_, and MeJA. Under salt and alkali stress, *TaMYC2* expression is induced in the roots, but it is inhibited in the stems. Drought and cold stress induced the expression of *TaMYC2* and increased antioxidant enzyme activities in the plants. At the same time, *TaMYC2* could also induce the expression of ROS scavenging-related and stress-responsive genes and increase the activity of antioxidant enzymes in plants, thus improving the ability of plants to resist stress.

## Supplementary Materials

The following supporting information can be downloaded at https://www.mdpi.com/article/10.3390/genes13020329/s1: Figure S1. Schematic diagram of the pCAMBIA1300-35S-sGFP vector; Table S1. Primers used in this study; Figure S2. RNA electrophoresis, quality assessment of Caucasian clover cDNA, and results of PCR amplification of the *TaMYC2* gene; Figure S3. Bioinformatic analysis of *TaMYC2* in Caucasian clover; Table S2. The CDS and encoded amino-acid sequence of *TaMYC2*.
