# Supplementary material for: The Caucasian Clover Gene TaMYC2 Responds to Abiotic Stress and Improves Tolerance by Increasing the Activity of Antioxidant Enzymes
Source: Genes (Basel). 2022 Feb 10;13(2):329. doi: 10.3390/genes13020329 (PMC8871790; doi:10.3390/genes13020329)
Supplement: Supplementary file 1 [file genes-13-00329-s001.zip › genes-1533819-supplementary.pdf]

Table S1. Primers used in this study.

Primer name	Primer sequence(5'-3')
TaMYC2-S	ATGCTTTCGTTCACTTCCG
TaMYC2-A	TCACCAATTTTGGAAAAAAAGA
TaMYC2-F	GCGTACTCTTACCGGCTTCA
TaMYC2-R	GCCGCTTCTCTTGTTCCAGT
RG-F	TAATGCACCGAATCCCTCGT
RG-R	GAATGCAGATGACCGGTTGG
TaMYC2-TE-S	CGGGGGACTCTTGACGAGCTCATGCTTTCGTTCACTTCCG
TaMYC2-TE-A	CATGTCGACTCTAGAGGATCCCCAATTTTGGAAAAAAAGAGCA
NtActin-F	CTATTCTCCGCTTTGGACTTGGCA
NtActin-R	ACCTGCTGGAAGGTGCTGAGGGAA
NtSOD-F	CTCCTACCGTCGCCAAAT
NtSOD-R	GCCCAACCAAGAGAACCC
NtCAT-F	AGGTACCGCTCATTCACACC
NtCAT-R	AAGCAAGCTTTTGACCCAGA
NtPOD-F	GCTGTTCGACGAGTTGTTAACAG
NtPOD-R	CTCTGGCTGAGTTGTTGTTGG
NtLEA5-F	TTGAATCTGGGGTTTTGGTT
NtLEA5-R	GGAAGCATTGACGAGCTAGG
NtERD10C-F	AACGTGGAGGCTACAGATCG
NtERD10C-R	GTTCCTCTTGGGCATGAGTT
NtERD10D-F	GAGGACACGGCTGTACCAGT
NtERD10D-R	GCGCCACTTCCTCTGTCTT
HYG-F	acggtgtcgtccatcacagtttgcc
HYG-R	ggaagtgcttgacattggggagttt

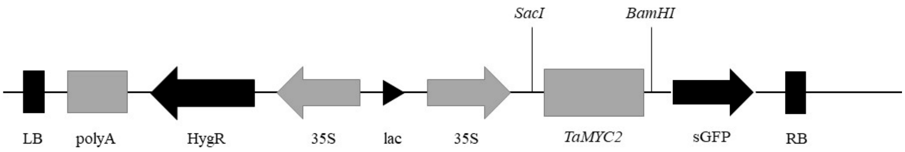

Figure S1. Schematic diagram of pCBMBIA1300-35S-sGFP vector.

Table S2. The CDS sequence and encoded amino acid sequence of *TaMYC2*.

Name	Sequence
CDS sequence	ATGCTTTCGTTCACTTCCGGCGTACTCTTACCGGCTTCAAATATGAAATCC<br>GGTGGTGGCGGTGGAGGTGGAGATTCTGATAATTCGGATCTAGATGTTTC<br>CGTCGTAAAAGAAGCTGACAGCGGCAGAGTACTGGAACAAGAGAAGCG<br>GCCACGAAAGCGAGGACGAAAACCGGCCAACGGAAGAGAAGAACCATT<br>GAATCACGTTGAAGCAGAAAGACAGAGAAGAGAAAAACTGAATCAAAG<br>ATTCTATGCTCTACGTGCAGTGGTTCCAAATGTTTCTAAAATGGACAAAG<br>CTTCACTTCTTGGAGATGCAATTTCTTACATAAATGAATTGAAATCAAAGC<br>TTCAAGGACTTGAATCATCCAAAGGTGAATTAGAGAAACAATTAGATACA<br>ACAAAGAAGGAACTTGAACTAGCAACCAAAAATCCAACAATTCCAATTC<br>CATTAGATAAAGAAATTGAACAAACAAGTTCTAAGTTGATTGATTTGGAT<br>ATAGATGTGAAGATTATGGGTTGGGATGCAGTGATTAGGATTCAATGTAGC<br>AAGAAGAATCATCCTGCGGCGAAGTTGATGGCGGCATTGAAAGAATTAG<br>ATCTTGAAGTGAATCATGCTAGTGTGTCTGTAGTGAATGATTTGATGATTC<br>AACAAGCTTCAGTTAACATGGGGAGTCAATTTTACACTAAGGAACAACTT<br>TCATCTGCTCTTTTTTTCCAAAATTGGTGA
amino acid sequence	MLSFTSGVLLPASNMKSGGGGGGGDSDNSDLDVSVVKEADSGRVLEQEKR<br>PRKRGRKPANGREEPLNHVEAERQRREKLNQRFYALRAVVPNVSKMDKAS<br>LLGDAISYINELKSKLQGLESSKGELEKQLDTTKKELELATKNPTIPIPLDKEI<br>EQTSSKLIDLDIDVKIMGWDAVIRIQCSKKNHPAAKLMAALKELDLEVNHA<br>SVSVVNDLMIQQASVNMGSQFYTKEQLSSALFFQNW

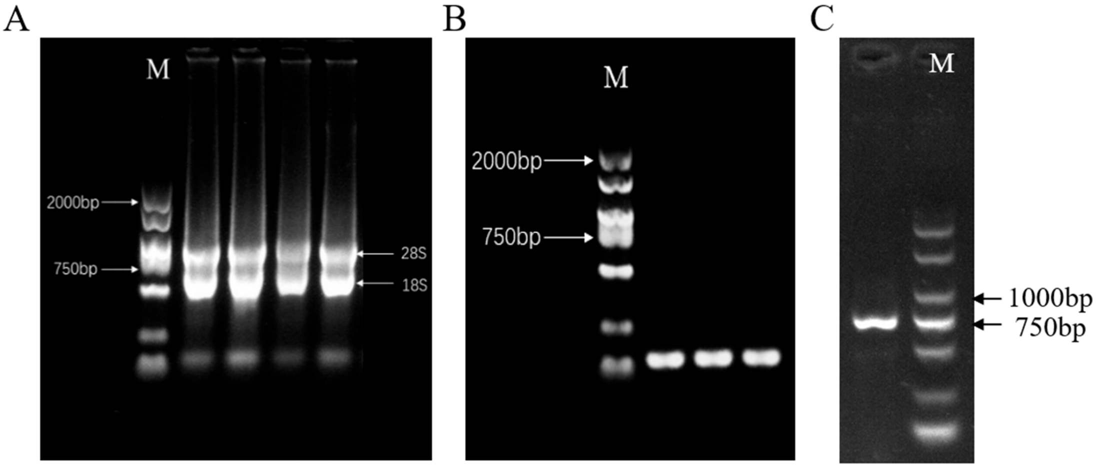

Figure S2. RNA electrophoresis (A)、cDNA quality assessment (B) of Caucasian clover and Results of PCR amplification of *TaMYC2* gene (C).

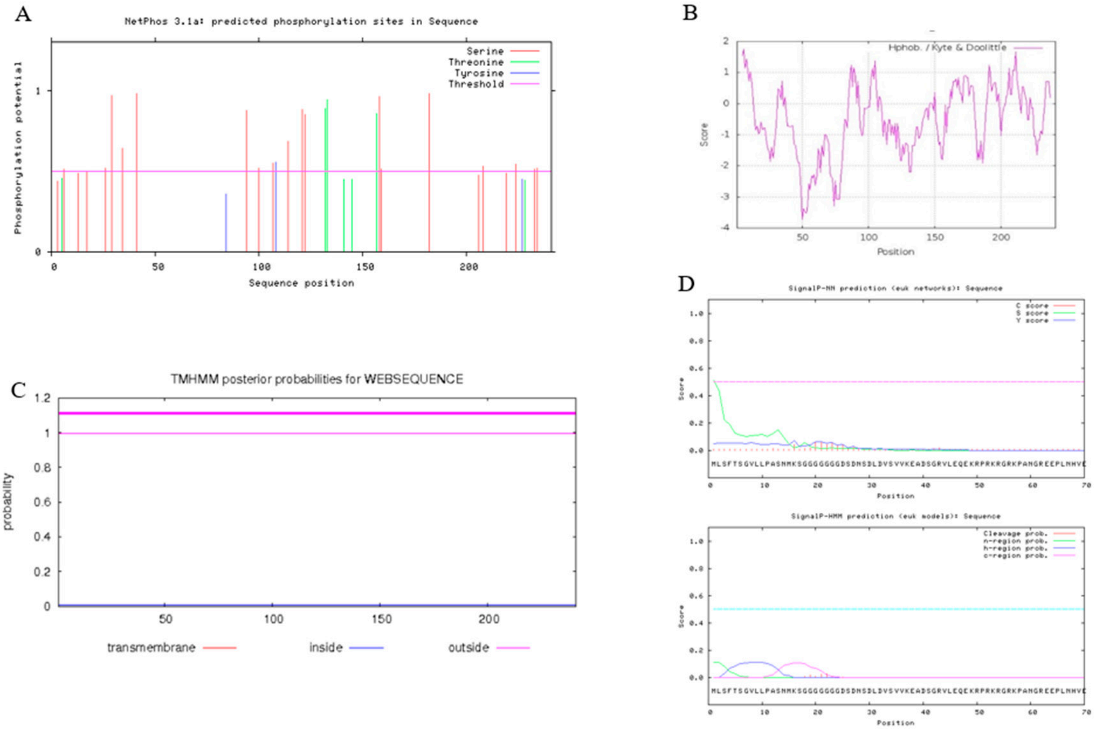

Figure S3. Bioinformatics analysis of *TaMYC2* in caucasian clover.

Notes: A: Prediction analysis of the phosphorylation sites. B: Protein hydrophilicity and hydrophobicity prediction. C: Prediction of the transmembrane domain. D: Protein signal peptide prediction.
